# New focus on basic research in China's advancement in science and technology

**DOI:** 10.1093/nsr/nwac014

**Published:** 2022-02-14

**Authors:** Mu-ming Poo

**Affiliations:** Scientific Director, CAS Center for Excellence, Brain Science and Intelligence Technology, China; Executive Editor-in-Chief of NSR

On 1 January 2022, a new version of the Law on China's Science and Technology (S&T) Advancement (‘科学技术进步法’) went into effect. This is the second revision of the Law, which was first implemented on 1 October 1993. The current revision is marked by extensive changes and additions that reflect China's rapid S&T development over recent decades and many new challenges facing China today. Many articles in the Law are elaborated with detailed descriptions of new strategies and plans for S&T advancements that are commensurate with China's economic prowess. Promotion of basic research stands out as one of the main goals for the future.

In the opening chapter on general provisions of the Law, four directions of future S&T advancement are laid out: exploring the world's scientific frontiers, meeting economic challenges, addressing major domestic needs and promoting people's health. These aim at economic and social progress together with sustainable development. Four pillars of the nation's strategic S&T force are defined: national laboratories, national research and development (R&D) institutions, top research universities and leading S&T enterprises. These pillars will provide leadership and support for innovation in the key areas and targeted directions of S&T. The Law sets the agenda for achieving a highly efficient, synergistic and open national innovation system, perfecting a healthy socialist market economy system that fully realizes market-directed and government-assisted S&T resource allocation, optimizing the sustainable flow of innovation elements and elevating systemic capability and efficiency with regard to achieving progress in targeted areas.

The government's support for basic research is highlighted in the second chapter of the Law. The principle is to combine the serving of the country's needs with free S&T exploration, via planning and the stable support of three types of research—major areas of basic science, frontier technology and public welfare technology. The Law stipulates a gradual increase in the government's contribution to overall S&T funding (with an increment higher than that of regular expenditure), a gradual increase in the percentage of GDP devoted to R&D, and a gradual elevation of the proportion of basic research funding within the total R&D expenditure. It also calls for industry's investment in basic research and society's contributions via gifts, donations and private foundation funding, with the government's financial support and tax benefits promised.

Complementing the support for basic research, the Law calls for a reform in the education system that links theoretical with practical education, and cultivates innovative capability, critical thinking and the spirit of pursuing truth based on facts. It also emphasizes the role of higher education institutions in scientific research, technological development and social services, as well as in cultivating high-level specialists with social responsibility, an innovative spirit and practical capabilities. New policies for cultivating S&T talents are detailed in the Law, and include elevating their social status, ensuring they have the appropriate environment for innovative activity (with appropriate evaluation criteria and incentives) and protecting their intellectual rights.

The foundation of basic research is ‘free exploration’, a recurring theme in the Law and many recent governmental pronouncements. This requires substantial reform in the S&T management system and institutional evaluation mechanism, in order to encourage and provide room for free S&T pursuits. Notably, there is a prominent emphasis on the fact that basic research needs to be guided by the goal of meeting the country's needs. This appears to be quite different from the notion held by many researchers that basic research entails free exploration without being influenced by predetermined goals, and that its outcome is often unexpected and serendipitous. But in fact, exploratory research as basic as pure mathematics and astronomy are often goal directed. Exploratory research is far from an aimless random walk, and major breakthroughs are often propelled by the desire to reach the goal of solving major S&T problems.

Impressive progress has been made in many S&T fields in China over recent decades, as reflected by the rapid rise in the total number and impact of research publications. However, the results of many basic research studies were limited to the stage ‘proof of principle’, and ended with publications in high-profile journals. The practical use of original concepts, on the other hand, requires a series of incremental advances in basic research and technical improvement that are less glorious and sometimes more difficult to achieve. There has been much recent attention, in the Chinese research community, on China's need to achieve ‘0 to 1’ breakthroughs, i.e. groundbreaking discoveries and inventions that result in paradigm shifts in various fields of S&T. Yet, equally important is the arduous task of ‘1 to 100’ advances that could truly bring practical benefits to society. By emphasizing goal-directed basic research, the new Law points to a well-defined new horizon for S&T advancement in China.

